# Different Patterns of White Matter Degeneration Using Multiple Diffusion Indices and Volumetric Data in Mild Cognitive Impairment and Alzheimer Patients

**DOI:** 10.1371/journal.pone.0052859

**Published:** 2012-12-31

**Authors:** Gilberto Sousa Alves, Laurence O’Dwyer, Alina Jurcoane, Viola Oertel-Knöchel, Christian Knöchel, David Prvulovic, Felipe Sudo, Carlos Eduardo Alves, Letice Valente, Denise Moreira, Fabian Fuβer, Tarik Karakaya, Johannes Pantel, Eliasz Engelhardt, Jerson Laks

**Affiliations:** 1 Alzheimer’s Disease Center – Institute of Psychiatry, Universidade Federal do Rio de Janeiro, Brazil; 2 Cognitive and Behavior Neurology Unit – Institute of Neurology, Universidade Federal do Rio de Janeiro, Rio de Janeiro, Brazil; 3 Radiology Service – Instituto de Neurologia Deolindo Couto, Universidade Federal do Rio de Janeiro, Procardíaco Hospital, Rio de Janeiro, Brazil; 4 Department of Psychiatry, Psychosomatic Medicine and Psychotherapy, Goethe-University, Frankfurt am Main, Germany; 5 Institute for Neuroradiology, Goethe-University, Frankfurt am Main, Germany; 6 Institute of General Practice, Geriatric Medicine, Goethe-University, Frankfurt am Main, Germany; “Mario Negri” Institute for Pharmacological Research, Italy

## Abstract

Alzheimeŕs disease (AD) represents the most prevalent neurodegenerative disorder that causes cognitive decline in old age. In its early stages, AD is associated with microstructural abnormalities in white matter (WM). In the current study, multiple indices of diffusion tensor imaging (DTI) and brain volumetric measurements were employed to comprehensively investigate the landscape of AD pathology. The sample comprised 58 individuals including cognitively normal subjects (controls), amnestic mild cognitive impairment (MCI) and AD patients. Relative to controls, both MCI and AD subjects showed widespread changes of anisotropic fraction (FA) in the corpus callosum, cingulate and uncinate fasciculus. Mean diffusivity and radial changes were also observed in AD patients in comparison with controls. After controlling for the gray matter atrophy the number of regions of significantly lower FA in AD patients relative to controls was decreased; nonetheless, unique areas of microstructural damage remained, e.g., the corpus callosum and uncinate fasciculus. Despite sample size limitations, the current results suggest that a combination of secondary and primary degeneration occurrs in MCI and AD, although the secondary degeneration appears to have a more critical role during the stages of disease involving dementia.

## Introduction

In recent years, novel methods of neuroimaging have enabled biological tissues to be investigated *in vivo* [1], [2]. One of the most useful of these techniques is diffusion tensor imaging (DTI), which is sensitized to the motion of water molecules as they interact within tissues, thus reflecting characteristics of their immediate structural surroundings [3], [4]. DTI signal is an indirect measure of various aspects of tissue integrity and may be influenced by myelin density, membrane intactness and possibly other fiber components [5], [6]. Diffusivity represented by the water motion in a particular region can thus be altered by ordered structures such as axonal tracts in nervous tissues [7]. Because diffusivity oriented by the fiber direction (anisotropic) is largely restricted in the gray matter (GM), an increase in anisotropic diffusion may correlate with myelin sheath content, thus being a valuable tool in assessing the microstructure integrity of white matter (WM) fiber tracts [8]. By sampling the diffusivity along multiple directs spaced on a sphere a representation of the ellipsoid can be computed [4], [8]. DTI uses measures derived from the eigenvectors that define the diffusion ellipsoid in each voxel [4]. Axial diffusivity reflects the diffusion coefficient along the principal eigenvector (λ1), whereas radial diffusivity indicates the average diffusion coefficients along the two axes perpendicular to λ1 Mean diffusivity (MD) is a measure of the total amount of diffusion within a voxel and is computed as an average of all three diffusion axes [4]. Finally, fractional anisotropy (FA) is a scalar value between zero and one and it is calculated from the eigenvalues (λ1, λ2, λ3) of the diffusion tensor [4]; this parameter indicates the degree of anisotropy of a diffusion process. Diffusion properties associated with multiple DTI measures can be interpreted as reflecting different structural contributions to WM integrity. The coherence of the orientation or directionality of water diffusion is reflected by FA, which is thought to provide an overall assessment of WM integrity [4] and is the most commonly used parameter in DTI studies [9]. MD depends on the density of physical obstructions such as membranes and the resultant distribution of water molecules between different cell compartments [6]. MD is a measure of overall gross diffusion and higher MD values point to increased diffusion and suggest tissue breakdown with increased brain water content [4]. Axial and radial indices provide more specific information about the integrity of axons versus their surrounding myelin sheaths: an increase in radial diffusivity has been linked with loss of myelin in animal studies of experimentally induced myelin loss [10], [11]; in contrast, a decrease in axial diffusivity has been reported in rodents and in the majority of DTI studies with humans, being associated with axonal swelling and fragmentation [11], [12].

Alzheimeŕs disease (AD) is the most prevalent neurodegenerative disorder that causes cognitive decline in old age [13]. The pathology of AD stems from the deposition of two proteins β-amyloid and tau [14], which leads to a loss of cortical neurons with consequent cerebral atrophy [15]. Even though cortical disease has been primarily considered in AD, increasing evidence from DTI has reported diffusivity changes in AD to WM degeneration and anatomical loss of connectivity which may be associated with different mechanisms, including axonal damage and demyelination [16]. It is hypothesized that myelin pathology leads to β-amyloid axonal damage in the WM tracts thus increasing the vulnerability to neurodegenerative processes which culminate in the clinical symptoms of AD [17].

Newly proposed criteria for AD and its preclinical states encourage the use of DTI and other biomarkers to enhance early diagnosis in those likely to develop cognitive disorders [12], but the clinical significance of diffusion indices deserves a more comprehensive understanding. Early DTI studies have employed FA alone or in combination with MD [9]. It has also been suggested in AD and MCI patients that an increase in radial and axial diffusivities may be more reliable markers of degeneration than FA, which is a function of the ratio of these diffusivities [18], [19] and may not be able to detect subtle changes in WM [19]. Furthermore, recent reports suggest that the sensitivity of radial diffusivity to the changes that occur in WM during normal aging is higher than that of axial diffusivity, possibly because the former is more closely associated with myelin breakdown [20]; conversely, other studies indicate that axial diffusivity increases are more useful in identifying early changes (e.g., axonal damage) in AD [12]. Because of these constraints, the investigation of multiple indices of DTI may help to develop a more comprehensively understanding of the landscape of diffusion changes in AD [18]–[21]. Another less explored topic is the association of brain volumetric changes observed in cortical and subcortical areas with DTI findings. Two main pathophysiological hypotheses leading to the WM damage have been discussing by the recent literature: according to the Wallerian degeneration model, degradation of WM microstructure can occur secondary to GM pathology resulting from the accumulation of aggregated hyperphosphorylated tau protein, deposition of β-amyloid [22], oligodendrocyte death and reactive gliosis [23]; conversely, the retrogenesis theory suggests that WM degeneration results from a pattern that is the reverse of myelogenesis with late-myelinating pathways being first affected by AD (specially in the neocortical projections), and early-myelinating pathways affected later in the disease [17], [24].

Among the voxel based methods, Tract-based spatial statistics (TBSS) [25] is a powerful technique for investigating WM microstructure. TBSS has additional advantages in comparison to conventional region of interest (ROI) oriented DTI, specifically a higher accuracy in differentiating between cerebrospinal fluid and GM and the possibility of aligning data onto a WM skeleton, thus minimizing the effects of misalignment [25]. Additionally a higher reliability and reproducibility of TBSS has been demonstrated by previous studies [18], [20], [26], [27].

The objectives of this study are twofold. First, we sought to describe and investigate the main DTI changes in subjects with different cognitive status using the FA, MD, radial and axial indices in order to characterize and compare the patterns of WM microstructural changes between these groups. Secondly, we investigate the underlying WM pathology in distinct brain areas using two complementary procedures: the interpretation of FA, MD, radial and axial diffusivity changes through the overlapping between those indices; the correlation between anisotropic values in regional WM tracts with volumetric findings in gray matter, WM, and whole brain.

We hypothesize that multiple indices of diffusion would be altered in the early stages of cognitive decline and that WM degeneration may follow a distinct and progressive pattern that becomes more pronounced and widespread in more severe disease states. Based on the recent literature [18]–[21], [28]–[30], we wanted to assess whether or not Wallerian degeneration and/or retrogenesis contributed to WM damage.

## Materials and Methods

### Ethics Statement

The study protocol was prepared in accordance with ethical standards laid down in the declaration of Helsinki and was approved by the Institute of Psychiatry from the Federal University of Rio de Janeiro ethics committee. Patients and controls signed a written consent following a full oral description of the study.

### Participants

Twenty three individuals with AD, 18 with MCI and 17 controls were recruited from a cohort regularly followed at the Alzheimeŕs disease Center of the Federal University of Rio de Janeiro from January 2007 to December 2009. Participants were submitted to a broad standardized clinical assessment including geriatric, neurological, neuropsychological and psychiatric exams to define clinical status. A panel consisting of three neuropsychiatrists with expertise in dementia research (FKS, CEA, EE) made clinical decisions, including the assignment of CDR rating. The cognitive assessment included the CAMCOG [31], Mini-Mental State Examination (MMSE) [32], semantic verbal fluency (animal category), Clock Drawing Test (CLOX1) [33], Trail Making Tests (TMT) A and B [34], and the 12-item Boston Naming Test [35].

Diagnosis of Mild Cognitive Impairment (MCI) amnestic (single or multiple domain) was established according to the Petersen criteria [36] as follows: (a) memory complaint corroborated by an informant, (b) objective memory impairment corrected for age, education and gender; (c) essentially preserved general cognitive function; largely intact functional activities, (e) not demented. To fulfill the item “b” of the Petersen criteria we assessed the memory performance in the Cambridge Cognitive Examination (CAMCOG) [31]. Because of educational heterogeneity in the sample, normative values suggested by a previous study with 292 elderly subjects in the Alzheimeŕs disease Center of the Federal University of Rio de Janeiro were adopted [37]. Subjects with cognitive performance 1.5 S.D. below the quartile values recommended by this study for age and education were included as MCI. All MCI individuals had an overall Clinical Dementia Rating (CDR) [38] of 0.5 and scored below 4 in the Pfeffer Functional Activities Questionnaire [39] and were included if they were subtype amnestic (single or multiple domain). AD patients met the diagnostic criteria of the Neurological Disorders and Stroke-Alzheimer disease and Related Disorders (NINCDS-ADRDA) working group [40]. Clinical diagnosis was reviewed periodically for a period of two years after the beginning of the study during which regular medical evaluations were performed, and cases that converted to any type of non-AD cognitive disorder were excluded. During the time of the study, patients` family members (or spouses) were evaluated by the same protocol as outlined above and were selected as healthy older controls if they fulfilled the criteria for this group.

Exclusion criteria for all participants were a history of seizures, major psychiatric disorder, motor and sensorial impairment, dementia, impaired thyroid function, abuse of alcohol or substance abuse or dependence. Clinical, structural and DTI data presented in this study were acquired at baseline and analyzed in 2011 at the Laboratory of Neuroscience (Dept. of Psychiatry) and the Institute for Neuroradiology of the Goethe-University, Frankfurt.

### Structural Image Protocol

MRI was conducted with a 1.5 T MRI machine (General Electric, Milwaukee, WI, USA). A T2-weighted fluid attenuated inversion recovery sequence (FLAIR) was acquired using the following parameters: TR = 10,000 ms; TE = 90 ms; TI = 2100; 20 slices; slice thickness = 5 mm/spacing = 1 mm, field of view (FOV) = 24; acquisition matrix = 256×192; 1NEX; total acquisition time = 2 min 40 sec. T1-weighted structural images were achieved with the following pulse sequence: TR = 500 ms, TE = MinFull, 20 slices; slice thickness = 5 mm/spacing = 1 mm, FOV = 24; acquisition matrix = 320×192, 2NEX, total acquisition time = 3 min 16 sec.

DTI scans were acquired using a gradient echo sequence with the following parameters: TR = 10000 ms, TE = 88.3 ms, acquisition matrix = 128×128, FOV = 30 mm, 29 transaxial slices, 25 encoding directions (1NEX, b = 1000 sec/mm2), one reference image (b = 0), slice thickness = 5 mm/spacing = 0, total acquisition time: 4 min 40 sec.

### Volumetric Measurements

#### Total brain volume, white matter, and gray matter volumes

Brain tissue volumes normalized for each subject´s head size were estimated with SIENAX [41], which is part of the FSL library [42]. SIENAX starts by extracting brain and skull images from the single whole-head T2-weighted images [43]. The brain image is then affine-registered to the MNI152 standard space [44], [45] using the skull image to determine the registration scaling; this is primarily in order to obtain the volumetric scaling factor, to be used as a normalization for head size. Next, tissue-type segmentation with partial volume estimation is carried out [46] in order to calculate total volume of brain tissue, including separate estimates of volumes of GM and WM.

#### Hippocampal volume

The right and left hippocampal volumes were automatically segmented using 3D T1-weighted volumes and fMRIB`s Integrated Registration and Segmentation Tool (FIRST) [47], part of FSL [48]. To perform segmentation, the input data were first transformed to the MNI (Montreal Neurological Institute) 152 standard space and a subcortical mask with a boundary correction method was applied to locate the structures. Hippocampal volumes were calculated using the FIRST label/structure correspondence with normalized values being computed by FSL stats.

### White Matter Hyperintensities

All FLAIR images were visually inspected by one investigator (CK) blind to any clinical data. WM hyperintensities burden was quantified with MRICron [49], following the procedures outlined by Berlow et al. [50]; a 2-step thresholding process was performed on all voxels containing WM hyperintensities by visual inspection being included; subsequently non brain voxels such as skull and eyes were removed after manual editing; the volume in milliliters was finally obtained by multiplying the identified voxels by voxels dimensions and the product was a normalized skull size using the SIENAX scaling factor. Parameters of WM volume estimation of the LADIS study [51] were employed to exclude cases of severe WM hyperintensities (>20 mm diameter and grade = 3); five subjects (3 females, 2 males) were therefore removed from the dataset. Excluded patients did not differ in age (F = 4.217, df = 11.001, *P* = 0.353), years of education (F = 2.535, df = 61, *P = *0.261) or gender (chi-square = 0.018, df = 1, *P* = 0.893).

### DTI Preprocessing

DTI Preprocessing and voxelwise statistical analysis of diffusion data were performed using FSL tools (FMRIB Software Library, Oxford, UK-http://www.fmrib.ox.ac.uk/fsl/). First, each subject´s data was checked by two investigators (GSA and AJ) in order to detect artifacts (spikes, motion) corrupted volumes which were subsequently removed from the dataset using an in-house script pipeline. Artifact correction has been demonstrated to increase the quality of DTI data [52] and can reduce the number of false positive results in the voxelwise analysis. Next, diffusion weighted images were corrected for motion and eddy current effects by coregistration to the brain extracted b = 0 image [FSĹs Brain Extraction Tool (BET)] [53]. The diffusion tensor was then calculated with the DTIFIT program (part of FSL) finally providing FA, MD [(λ1 + λ2 + λ3)/3 ], radial [(λ2 + λ3)/2] and axial (λ1) diffusivity maps. Those maps were subsequently used in the TBSS analysis. Most processing steps were performed automatically using an in-house script pipeline (MRIST - M R Imaging and Spectroscopy Toolbox, Institute for Neuroradiology, Goethe-University, Frankfurt am Main, Germany).

### DTI Statistical Analysis with TBSS

TBSS scripts were used to perform a non-linear registration that aligned each FA image to every other one. This created a calculation of the amount of warping needed for the images to be aligned. The most representative image was determined as the one needing the least warping for all other images to align to it. This target image was affine-aligned into 1×1×1 mm^3^ MNI152 standard space. Each FA image was then transformed into MNI152 space by applying their respective nonlinear transforms to the target and then the affine transform to MNI space. The aligned FA images were averaged to create a mean FA image which was thinned using an FA skeletonization program (threshold FA value of 0.2). This identified all fiber pathways consistently across all subjects, FA data were then projected onto the mean FA skeleton that is common to all participants [25].

A standard approach with the simple permutation function (randomize, v 2.1) in FSL was used on the skeletonized data to calculate voxel wise differences between AD, MCI and controls. Voxel wise statistics were carried out using a General Linear Model (GLM) and voxelwise group comparisons were performed using simple two sample T-test [54]. As the mean years of age tended to be higher in AD and there were statistically more females in the control group (Table 1), these two variables were input as covariates in the voxel wise analysis. The number of permutations was set to 5,000; the level of significance was adopted at *p*<0.05 level and corrected for multiple comparisons using the “2D” parameter settings with threshold-free cluster enhancement (TFCE), a method which avoids using an arbitrary threshold for the initial cluster-formation [55]. In order to characterize the different patterns of neurodegeneration, overlapping areas between FA and MD and FA and radial diffusivity were identified and their clinical meaning discussed.

**Table 1:** Demographic and clinical characteristics of the sample groups.^1^

	CN (n = 17)	MCI (n = 18)	AD (n = 23)	F/χ^2^(P)
**Gender (F/M)**	14/3	9/9	10/13	6.530 (0.038)
**Age**	71.18±8.06	72.83±6.51	76.35±6.73	2.823 (0.068)
**Education (years)**	10.41±3.52	7.11±4.75	9.04±5.17	2.272 (0.113)
**CDR**	0.00±0.00	0.39±0.27	1.39±0.66	53.741 (<0.001)*
**MMSE**	28.35±1.41	26.22±3.42	20.35±5.91	19.308 (<0.001) †
**Pfeffer**	0.06±0.24	1.44±1.46	16.83±7.18	84.285 (<0.001) †
**Clock Drawing Test**	13.88±1.05	12.06±2.63	8.91±3.90	14.754 (<0.001) †
**Verbal Fluency**	16.24±4.18	13.61±4.53	9.82±5.10	9.374 (<0.001) §
**Memory-CAMCOG** 2	21.58±2.69	16.94±4.57	8.72±5.51	40.64 (<0.001)*
**Boston Naming Test**	11.24±0.90	10.11±1.23	9.23±1.95	8.796 (<0.001)Δ

Values are displayed as mean ± SD. M = male, F = female. CDR = Clinical Dementia Rating Scale; MMSE = Mini-mental state examination; CAMCOG = Cambridge Cognitive Examination; CN = Cognitively Normal; MCI = Mild Cognitive Impairment; AD = Alzheimer Dementia;

1 Bonferroni correction;

2 Composite score with memory items from CAMCOG and MMSE.

Post hoc analysis with Bonferroni correction:

* AD vs CN, *P*<0.01; AD vs MCI, *P*<0.01; MCI vs CN, *P*<0.05;

† AD vs CN, *P*<0.01; AD vs MCI, *P*<0.01; MCI vs CN, *P*>0.05;

§ AD vs CN, *P*<0.01; AD vs MCI, *P*<0.05; MCI vs CN, *P*>0.05;

Δ AD vs CN, *P*<0.01; AD vs MCI and MCI vs CN, *P*>0.05.

Following analysis with randomize and the creation of the WM skeleton, ROI areas were created using a semi-automated procedure that incorporated 20 structures identified probabilistically by the Johns Hopkins University WM tractography Atlas [56]–[58]. These structures were: anterior thalamic radiation Left/Right (L/R), corticospinal tract L/R, cingulum (cingulate gyrus) L/R, cingulum (hippocampus) L/R, forceps major, forceps minor, inferior fronto-occipital fasciculus L/R, inferior longitudinal fasciculus L/R, superior longitudinal fasciculus (SLF) L/R, uncinate fasciculus (UF) L/R, SLF temporal part L/R. An intersection between statistical significant TFCE images threshold at 5% and each atlas ROI was done in order to create a mask that belongs to a particular anatomical tract. FSL stats computed single subject FA values using the masks (intersection ROIs) and individual FA images.

### Statistics

The Kolmogorov-Smirnov test assessed the distribution of curve for DTI and volumetric variables. As a substantial deviation from normality was not observed, parametric tests were used for whole skeleton comparisons and correlations with brain volumetric variables. The demographic and clinical data from the three groups were compared by one-way analysis of variance (ANOVA). To compare gender differences, the χ^2^ test was applied. The correlation between FA values within masks and volumetric variables was assessed with Pearsońs rank correlation. A *p* value <0.05 was adopted as statistically significant. All statistical analyses were performed with SPSS statistical package version 15.0.

## Results

Table 1 illustrates the demographic and cognitive characteristics of the sample groups. The gender distribution for the MCI and AD groups did not show significant differences.

The CDR score was 0 for controls, 0.5 for MCI subjects and for AD patients 1 (n = 16) 2 (n = 5) and 3 (n = 2).

### Structural Brain Differences

The multivariate analysis is reported in the Table 2. The normalized GM and right and left hippocampal volumes were significantly different among the groups. A post hoc Bonferroni correction found that the AD group had significantly lower right and left hippocampal volumes than the MCI and control groups. GM volume was significantly lower in the AD than in the control group. There were no significant differences between groups with respect to normalized WM, intracranial volume or WM hyperintensities burden.

**Table 2:** Brain volumetric comparisons with normalized values.

	CN (n = 17)	MCI (n = 18)	AD (n = 23)	F (P)
**Grey Matter^1^**	655,531±157,698	567,854±176,335	473,575±171,231	5.725 (0.006)*
**White Matter^1^**	742,875±100,817	685,995±207,756	779,344±203,226	1.344 (0.269)
**Hippocampus right^1^**	3,886±0,534	3,903±0,703	3,191±0,865	6.426 (0.003)^†^
**Hippocampus left^1^**	3,957±0,679	3,770±0,563	3,109±0,704	9.180 (0.000)^§^
**Intracranial Volume^1^**	1398,406±198,649	1253,850±156,211	1252,919±308,455	2.216 (0.119)
**WMH^2^**	8.200±6.661	8.338±4.394	10.919±6.694	1.300 (0.281)

WMH = white matter hyperintensities; CN = Cognitively Normal; MCI = Mild Cognitive Impairment; AD = Alzheimer Dementia; volume expressed in mm^3^ (1) and ml (2).

Post hoc analysis: *AD vs CN, *P* = 0.004; ^†^ AD vs CN, *P* = 0.013; ^†^ AD vs MCI, *P* = 0.009; ^§^ AD vs CN, *P* = 0.001; ^§^AD vs MCI, *P* = 0.012; values are displayed as mean ± standard deviations;

### DTI Differences

#### Whole skeleton results

**Figure 1:**
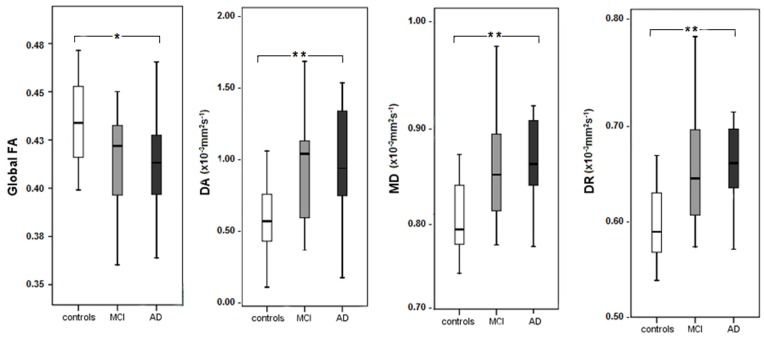
Distribution of diffusion tensor MRI indices for the global WM ROI in controls, MCI and AD patients. Multivariate Analysis reported significant differences between groups for FA (F = 4.649, df = 2, *P*<0.05), axial diffusivity (F = 5.610, df = 2, *P*<0.05), MD (F = 6.821, df = 2, *P*<0.005) and radial diffusivity (F = 7.131, df = 2, *P*<0.005). *P<0.05, **P<0.01, corrected for multiple comparisons using Bonferroni correction.

The distribution of diffusion tensor MRI indices for the global WM ROI in controls, MCI and AD is shown in the Figure 1. The ANOVA analysis found significant differences between groups for all indices. FA was lower in MCI relative to controls, while FA values in AD were lower than those in MCI, indicative of a progressive worsening of white matter integrity. MD, axial and radial diffusivities were significantly increased in the AD group relative to controls. In comparison with controls, MCI subjects showed a non significant trend toward lower FA values (P = 0.068) and increased MD (*P* = 0.068) and radial diffusivity (*P* = 0.057) values.

### Regional Area Results with TBSS Images

#### FA results

**Figure 2:**
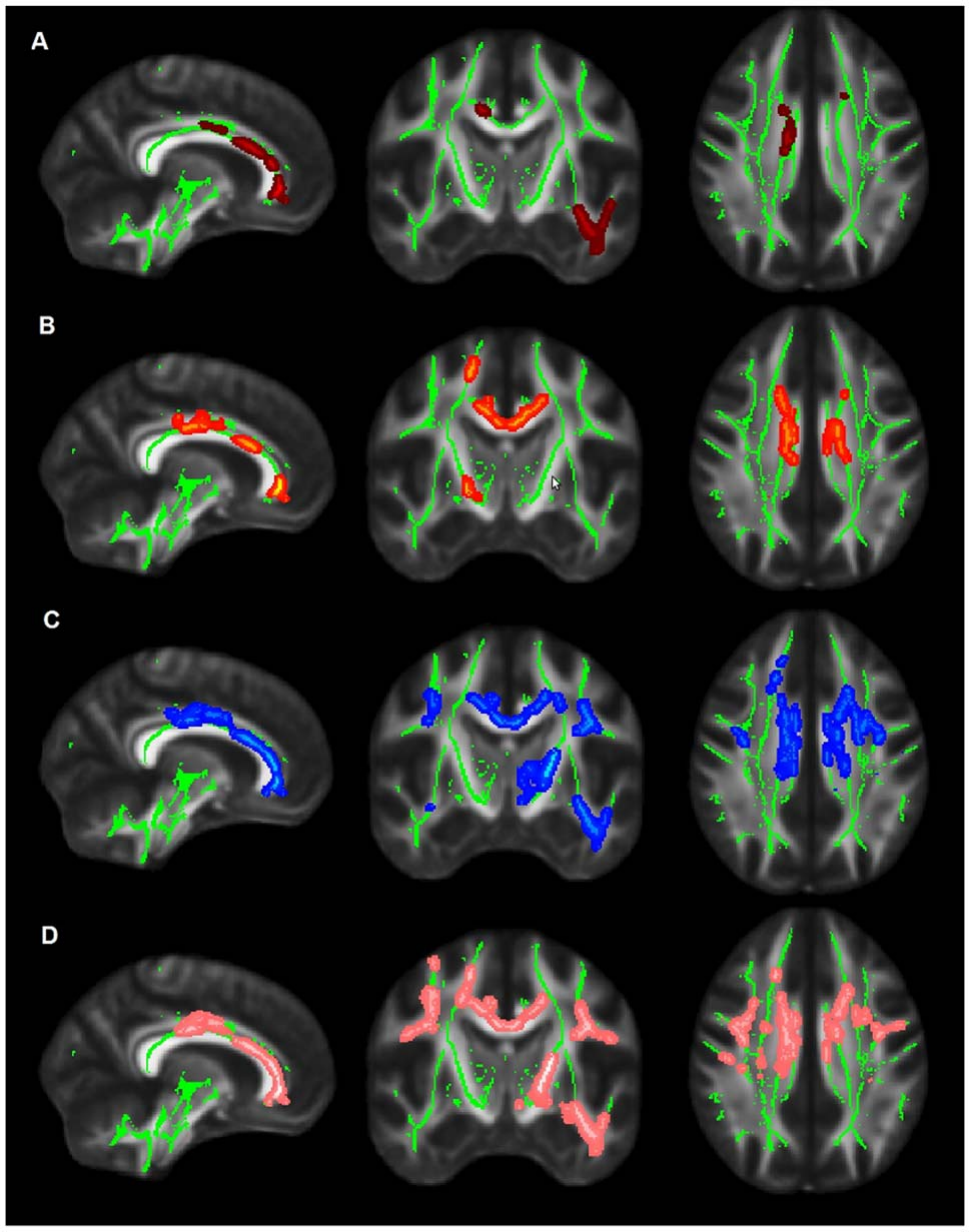
TBSS maps showing voxelwise comparisons between patients and controls. The mean FA skeleton (green voxels) projected on the FMRIB template brain. Low FA in AD patients in is shown in dark red (A); low FA in MCI is shown in yellow-red (B); high MD in AD is depicted in blue (C) and high radial diffusivity in orange.

A comparison of the AD and control groups revealed low FA (Figure 2) predominantly in the anterior-inferior segment of the brain, specifically in the following tracts: genu and body of the corpus callosum, right cingulate gyrus (anterior and middle portions), left cingulate gyrus (anterior portion), anterior corona radiata (bilateral), inferior occipital frontal fasciculus (bilateral), UF (bilateral), right superior occipital frontal fasciculus, inferior longitudinal fasciculus (bilateral), and left external capsule.

Similarly to the AD group, MCI patients exhibited extensively decreased FA compared to controls in the anterior-inferior portion of the brain (Figure 2); although FA changes predominated slightly in the left hemisphere. Lower FA was identified in the genu and body of the corpus callosum, anterior corona radiata (bilateral), right and left cingulate gyrus (anterior and middle portions), right anterior limb of the internal capsule, right cortico-spinal tract, external capsule (bilateral, but more widespread in the right hemisphere), UF (bilateral, anterior portion), right inferior occipital frontal fasciculus and left superior corona radiata.

### MD Results

Compared to controls, AD patients exhibited higher MD in the following tracts (Figure 2): genu and body of the corpus callosum, anterior, middle and ventral posterior segments of the cingulum bundle, forceps minor (bilateral), temporal part of the SLF(bilaterally), anterior limb of the internal capsule (bilateral, but predominant in the left side), right inferior longitudinal fasciculus, UF (bilateral), inferior fronto- occipital fasciculus (bilateral), anterior thalamic radiation (bilateral), anterior corona radiata (bilateral), left external capsule, parafascicular nucleus/centromedian nucleus (thalamus).

No MD differences were identified in the MCI-controls or MCI-AD group comparisons.

### Radial Diffusivity Results

Radial diffusivity followed MD increases in the AD group relatively to controls. The areas showing radial diffusivity increases in the AD group were genu and body of the corpus callosum, cingulate gyrus (bilateral, anterior and middle segments), anterior limb of the internal capsule (bilateral), external capsule (bilateral), SLF temporal part (bilateral), left inferior longitudinal fasciculus, left corticospinal tract, left inferior and superior temporal gyrus, forceps minor (bilateral), UF (bilateral), left anterior corona radiata, SLF (bilateral), anterior thalamic radiation (bilateral) and right post-central gyrus white matter.

No significant radial diffusivity differences were identified between the MCI-controls or MCI-AD group comparisons.

### Axial Diffusivity Results

No axial diffusivity differences in the voxelwise analysis were found for group comparisons.

### Regions of Overlapping Diffusion Findings

**Figure 3:**
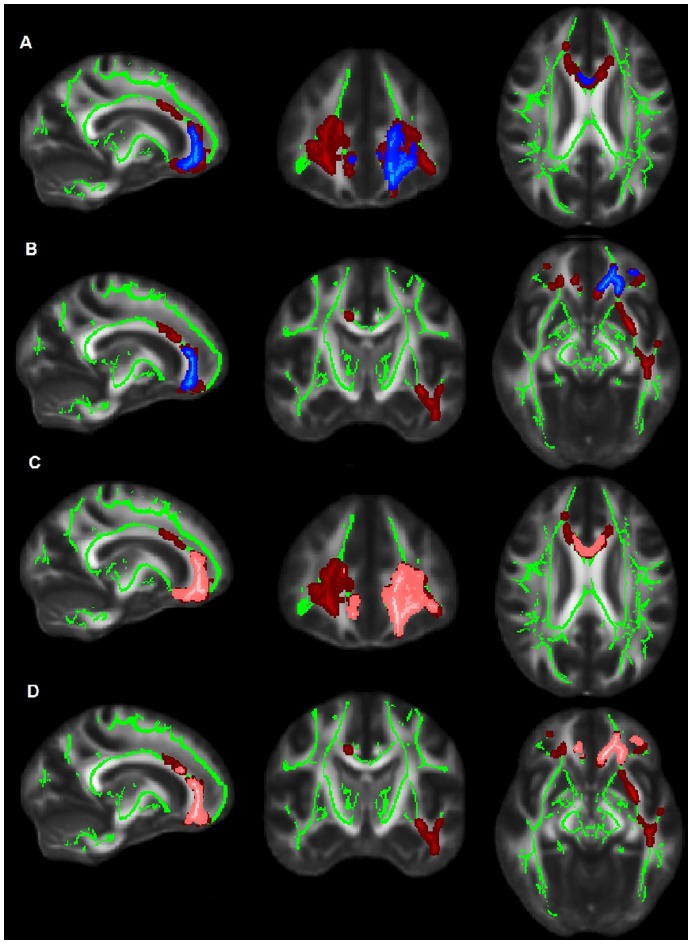
Overlapping WM regions between controls and AD for specific DTI indices. Areas of overlap between low FA (red) and high MD (blue) suggest gross tissue loss and are depicted in the upper panels (A) and (B); FA decreases (red) overlapping with radial diffusivity increases (orange) are shown in the lower panels (C) and (D) and suggest areas of myelin damage.

The interpretation of overlapping indices has not been comprehensive [59] and is based largely on animal models studies [60]; relatively few human studies have examined these overlaps with equivocal findings [18], [20], [26], [61]. Nevertheless these studies have been provided additional insights into the understanding of cascade events associated with AD process. According to current models [12], the combination of low FA-high radial diffusivity is indicative of myelin damage [12]. Conversely, the overlap high MD low-FA may reflect water accumulation and gross tissue loss [12]. Overlapping changes in diffusion indexes are depicted in the Figure 3.

The FA↓, MD ↑overlap (Figure 3 a, b), which may indicate diffusion changes due to gross tissue loss, was found in small areas comprising the left anterior corona radiata, the left cingulate gyrus and the genu of the corpus callosum (left portion). Diffusion changes due to possible myelin damage (i.e. FA↓, radial diffusivity↑, Figure 3 c, d) were observed bilaterally but predominantly in the left hemisphere; these areas included parts the corpus callosum (left and right frontal tracts, the genu and the body) and the left anterior corona radiata.

### Voxelwise Analysis Adjusting for GM Atrophy Differences between Groups

**Figure 4:**
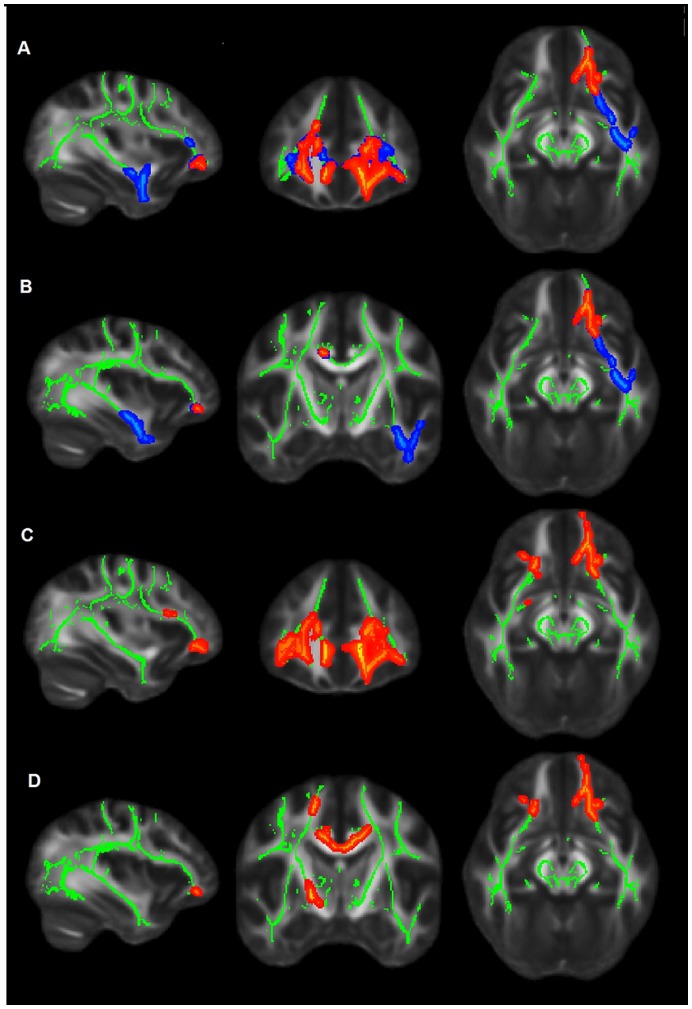
FA results adjusting for GM atrophy differences between groups. Upper panels (A) and (B): TBSS maps show low FA in the AD group in comparison to controls. The figures results from two GLM models designed for group comparisons. The model 1 included age and gender as covariates and is represented by the sum of blue and red voxels. The model 2 added as covariate the GM volume to the model 1 and is identified as red voxels. Red areas representing the remaining voxels after controlling for the GM atrophy can be seen mostly in the left hemisphere. They include WM tracts such as the left anterior corona radiate and the left and right corpus callosum (A) and the right cingulate gyrus (B). For a detailed description see the results section. Lower panels (C) and (D): TBSS maps show low FA in the MCI group relatively to controls. Note that statistical significant areas resemble those from the AD-control contrast seen before in the model 2 (upper panel).

TBSS maps resulting from two different GLM models (1 and 2) are presented in the Figure 4 (see related text for further explanation). After correction for the effect of GM atrophy (model 2), the following WM tracts remained statistically significant (with lower FA in the AD group): the left anterior corona radiata, left inferior fronto-occipital fasciculus (IFOF), bilateral frontal corpus callosum (that connect the frontal areas), and a small segment of the cingulate gyrus (in the right hemisphere) and corpus callosum (in the left hemisphere). Conversely, the following WM tracts associated with higher GM atrophy in the AD group were: left inferior longitudinal fasciculus (including its occipital fibers) and left inferior temporal gyrus.

### Correlations between FA Values and Brain Structural Data

Table 3 depicts correlations between FA values in the statistically significant ROIs and structural variables in MCI and AD patients. In the MCI group, FA correlated positively with the WM volume in the majority of ROIs: a) in both hemispheres with the anterior thalamic radiation, cingulate gyrus, inferior fronto-occipital fasciculus, UF; b) in the right hemisphere with the SLF (SLF) and the SLF temporal part; c) in the left hemisphere with the hippocampal cingulum; d) forceps minor. In the AD group, neither WM nor GM exhibited statistically significant correlations with anisotropic values. In the left hemisphere WM hyperintensities burden correlated inversely with FA values in the following tracts: anterior thalamic radiation (r = - 0.414, *P*<0.05), SLF (r = - 0.469, *P*<0.05) and UF (r = −0.416, *P*<0.05). For both MCI and AD no significant correlations were found between FA and hippocampal volumes.

**Table 3:** Correlation^1^ between FA values and Brain Volumetric variables from the voxelwise ROIs.

ROI (FA values)^1^	Volume^2^			
	White Matter		Gray Matter	
	MCI	AD	MCI	AD
Anterior thalamic radiation (L)	0.566**	−0.001	−0.628**	0.288
Anterior thalamic radiation (R)	0.726**	0.002	−0.525*	0.256
Cingulate Gyrus (L)	0.653**	0.239	−0.448	0.207
Cingulate Gyrus (R)	0.635**	0.119	−0.439	0.153
Cingulum hippocampus (R)	−0.422	NA	NA	NA
Cingulum hippocampus (L)	0.170	NA	0.546*	NA
Forceps Minor	0.734**	0.142	−0.567*	0.283
IFOF(L)	0.506*	−0.053	−0.483*	0.328
IFOF(R)	0.724**	−0.011	−0.596**	0.213
ILF(L)	−0.134	NA	0.116	NA
SLF (L)	0.124	0.022	−0.272	0.250
SLF(R)	0.505*	−0.054	−0.300	0.047
SLFtemporal part (L)	0.419	−0.111	−0.541*	0.281
SLFtemporal part (R)	0.478*	−0.034	−0.237	0.033
Uncinate Fasciculus (L)	0.509*	−0.074	−0.505*	0.321
Uncinate Fasciculus (R)	0.692**	−0.003	−0.696**	0.218

Note - Values are Pearson Correlation coefficients; *statistical significance at level <0.05 (*) and <0.01 (**); CN = Cognitively Normal; MCI = Mild Cognitive Impairment; AD = Alzheimer Dementia; NA = not assessed because WM tract was not statistical significant; R = right; L = left; IFOF = inferior fronto-occipito fasciculus; ILF = Inferior longitudinal fasciculus; SLF = superior longitudinal fasciculus.

1 FA values extracted from voxelwise region of interests (ROIs); for MCI the ROI from the contrast MCI versus controls (controlled for age and gender); for AD the ROI from the contrast AD versus control (controlled for gender, age and gray matter).

2 Volume in mm^3.^

## Discussion

The current results show widespread FA changes in patients with MCI and AD when compared to healthy elderly, particularly for tracts located in the prefrontal cortex and inferior frontal lobe. Extensive changes were also found for MD and radial diffusivity in the AD group. DTI was analyzed in combination with brain volumetric analysis and suggest that the development of cognitive decline may be associated both by the retrogenesis theory and by Wallerian degeneration because there is a combination of primary white matter damage and secondary damage due to cortical atrophy. These findings are consistent with previous work [18], [20], [21], [26], [62] and underline the higher sensitivity of multiple indices of DTI in comparison with single FA measurements to detect microstructural changes in WM.

### Fractional Anisotropy Findings

The FA regions found to be significantly affected in both MCI and AD included the anterior portion of the corpus callosum (body and frontal fibers), the anterior cingulate, the inferior fronto-occipito fasciculus and the anterior corona radiata. Current findings are in line with previous reports, which have found FA decreases in both AD and MCI patients in the corpus callosum, cingulum fibers, prefrontal cortex [16], [63], [64]; significantly lower FA in the UF in the AD [65] and in the frontal callosal fibers in the MCI group [66] in comparison to controls have already been reported. The present findings support that in a preclinical stage of dementia widespread changes in FA are associated with WM primary atrophy. Conversely, no significant differences in DTI indices were found between AD and MCI, a finding consistent with recent TBSS-based investigations [16], [20], [26], [67].

FA overlaps were clearly observed between MCI and AD. The areas of significant lower FA in AD relative to control overlap to a large extent with the anatomical distribution of FA decreases in MCI relative to control. As previously reported by some studies [16], [64], these results suggest a continuum for anisotropic changes over pre and dementia stages, with WM changes becoming progressively more evident as the cognitive level declines.

Similarly to our results, FA overlaps were previously described for the cingulum [64], [68], frontal corpus callosum [68] and UF [27], [69]. Moreover results suggest that for WM tracts of particular importance in AD, the patterns of neurodegeneration may follow an anatomical gradient, with more severe disease states showing more widespread pathology. This was observed in the UF, largely compromised in AD while in MCI patients DTI changes were more restricted to the frontal lobe. The UF connects the subgenual cortex to the inferior temporal lobe [70] and there is equivocal evidence for its involvement in pre-symptomatic AD, with DTI changes being reported by some TBSS studies [26], [27] while others found no changes [20], [67]. The UF is a long association tract with prominent connections to the fronto-orbital cortex, amygdala, temporal lobe and the subgenual region of hippocampus [71]. It has been linked to the episodic memory network functioning [72]–[75]. In the current results MCI and control subjects had no statistical differences in GM atrophy and the voxelwise analysis revealed a pattern of damage in UF quite similar to the AD-controls comparison in the model 2 (Figure 4). These results suggest that damage to the UF follow an anterior to posterior gradient which may be associated with the retrogenesis hypothesis in the early stages of neurodegeneration, when there is still no large scale gray matter atrophy. In later stages of the disease, when dementia is already manifest, hippocampal atrophy can further compromise the parts of the UF that lie within the temporal pole [18]. It is plausible to hypothesize that within the UF, retrogenesis is the first mechanism of damage followed by the Wallerian degeneration. However caution is needed with the interpretation of our data, since there is some evidence showing that in MCI hippocampus atrophy may contribute to UF changes [76].

### Mean Diffusivity and Radial Diffusivity Findings

Significant and widespread increases in MD and radial indices were seen in the AD group. All DTI changes were found in the medial prefrontal cortex and temporal lobe of the brain. Similar changes as reported here for radial diffusivity and MD were found in other studies with multiple DTI indices [18]–[21], [62]. Diffusivity changes in the corpus callosum of AD patients (Figure 3) are in agreement with previous reports showing high MD [77] and radial diffusivity [28], and low FA [28]. An increase in absolute diffusion indices (radial diffusivity and MD) was observed in the anterior and middle sections of the cingulate bundle in AD patients.

Our findings for the non FA maps (Figure 2) are in agreement with previous works [20] and show that MD and radial diffusivity increases may coincide with FA reductions, but also can be found in more extensive areas than FA decreases.

### Axial Diffusivity Findings

An increase in axial diffusivity as measured by a global mask was seen in Alzheimer patients in comparison with controls [(F = 5.610, df = 2, *P* = 0.005) (Figure 1)]. However the voxel wise analysis for this parameter produced a borderline significant result for the AD-controls contrast. The absence of statistical significant voxels in regional WM tracts may rely on the small sample size of the groups. An alternative explanation may be related to the complexity and dynamic of changes regarding the axial diffusivity index, as pointed by previous literature [26], [78]–[80]. When possible myelin damage occurs, axial diffusivity may be initially decreased. In later stages, this decrease may be followed by increases in this parameter as cellular debris is cleared by microglia [78], [79].

### Findings Related to Overlapping between Different Indices

Our findings show that in the left anterior cingulum and left genu and body of the corpus callosum areas of gross tissue loss (low FA-high MD) coincided to a large extent with regions of myelin damage as indicated by low FA and high radial diffusivity. The meaning of DTI changes of the anterior segment of corpus callosum (which includes genu and anterior body) has been highlighted in recent investigations with pre-clinical AD [29], [81]–[83]. The anterior portion of this bundle is responsible for the inter-hemispheric connection between the prefrontal association cortices and is implicated in monitoring information in working memory and in the active retrieval of information from posterior cortical association areas [82], [84], [85]. Contrasting with posterior corpus callosum atrophy, generally more associated with Wallerian degeneration, the anterior atrophy of these tracts has been reported as closely related to myelin breakdown [82]. Our findings for overlapping DTI indices in the AD group (Figure 4) suggest therefore that the underlying pathology of anterior corpus callosum is related to myelin breakdown. Di Paola et al. [82] have shown that myelin breakdown is a key mechanism in Alzheimeŕs Disease and according to the retrogenesis hypothesis it may affect late myelinating fibers [86] such as the UF, limbic pathways and callosal fibers [87]. Some authors postulate that AD begins with damage to myelin [17]. This process may involve amyloid and tau accumulation arising as byproducts from homeostatic repair mechanisms that are activated by this demyelination process [17].

### Findings Related to the Localization of Diffusion Changes

Diffusion changes in both MCI and AD patients were predominantly distributed in the prefrontal cortex, cingulate gyrus and corpus callosum, while in the AD group the left temporal was also compromised. Even though discrepancies in the literature exist, with some DTI studies with AD patients reporting WM changes in anterior regions [88]–[90], while others found a greater occurrence of posterior changes [62], [91], a different approach to the interpretation of diffusion changes has been proposed by the recent literature [87]. In this perspective, the characterization of WM degeneration pattern in AD should be more focused on the prediction of WM changes in vulnerable late-myelinating fiber pathways, rather than looking for anterior versus posterior gradients of changes [21], [87].

### Correlation of Regional DTI Changes with Structural Data

In MCI patients, primary atrophy of WM was the main structural variable associated with diffusion changes in the anterior thalamic radiation, cingulate gyrus, forceps minor, SLF and UF. These correlations endorse the results of voxelwise analysis (Figures 3 and 4) in which late myelination fibers are more vulnerable to primary pathological modifications on pre-dementia stages [17].

As expected, we also found a positive correlation between FA in the hippocampal cingulum and GM volume. Hippocampal fibers connect the prefrontal and parahipocampal cortices [92], [93]. Disruption between hippocampal cingulate and memory brain networks has been implicated in the development of cognitive decline, as outlined by MRI and functional studies [9] and may be associated with early atrophy of the hippocampal cortex, as demonstrated by previous studies supporting the Wallerian degeneration model [9].

In AD patients, global WM hyperintensities burden correlated with low FA in several tracts, particularly in the anterior thalamic radiation, SLF and UF. These lesions may often reflect small vessel pathology but are also frequently observed in AD. Our results support the view that frontal WM hyperintensities contribute to the clinical syndrome of AD. However the regional pattern of WM burden is still controversial, with previous reports showing a higher vulnerability for different areas such as the forceps minor [94]; likewise it is still unclear how the distribution of these lesions could interact with Aβ pathology in AD [95] and further studies are needed to elucidate the interaction of WM macrostructural changes and AD pathology.

Our study has a number of limitations that deserves discussion. Firstly the interpretation of the results is limited since there was no follow up on the DTI changes. There was a large SD of the MCI group for MD, axial and radial indices (Figure 1) and widespread FA changes were found in both AD and MCI, but and surprisingly, they were found to a greater extent in MCI in the left forceps minor and the right putamen. The reason for such discrepancies is not clearly understood but might reflect the heterogeneity in this cohort. Our conclusions are also restricted by the small sample size which prevented further comparisons between amnestic single domain and multiple domain MCI groups. Additionally, some MCI subjects of the current study can be already considered pre-clinical AD patients and may already show similar increases in MD, axial and radial diffusivities as those found in the AD group. According to the new diagnostic criteria (not yet validated for clinical studies) MCI due to AD has been considered a high risk group for conversion to AD and a highly accurate categorization is possible by combining positron emission tomography and lumbar puncture [96]. Because these instruments were not part of the study protocol, categorization between MCI converters and non converters could not be established. Future studies need to integrate DTI indices to other biomarkers in preclinical dementia. The interpretation of FA results has been questioned more recently, especially in relation to its specificity as a biological marker of disease progression. Some authors have suggested that FA should not be interpreted alone, because this index lacks sensitivity when diffusion changes proportionally along all three eigenvectors [18]. Finally we used a 1.5 Tesla machine, what might have limited the accuracy of our findings. Nevertheless, studies with a similar MRI technology reported diffusions changes in many analogous WM tracts as ours [63]–[65]; recent evidence with probabilistic tractography, which allows to trace pathways from crossing-fiber regions, also give support to our findings, by evidencing that the SLF, UF, corpus callosum (entire extension) and cingulate bundle are the most affected WM tracts in AD [80].

There is a marked diffusion changes in the UF and frontal callosal fibers in MCI and AD patients favoring the retrogenesis model as a primary mechanism of degeneration followed by a Wallerian degeneration over the course of the disease. We found that was a degree of both direct white matter pathology as well as a degree of GM atrophy. A global picture of the current results for late myelinating fibers is more likely to sustain the retrogenesis hypothesis. Nevertheless, evidence supporting the Wallerian Degeneration theory was also found, since our results showed that in demented individuals DTI changes reflected progressive changes in GM atrophy namely in the temporal lobe. Therefore neurodegeneration patterns of DTI changes may vary in conformity with cognitive status, being independently associated in MCI subjects with WM microstructural changes of the prefrontal cortex and inferior frontal lobe while on later stages WM pathology may be more strongly associated with GM atrophy.

Our results indicate that an integrative approach of DTI and volumetric analysis may give insights to the investigation of WM landscape pathology in AD. Further research oriented on the anatomical specificity of microstructural abnormalities of WM fibers will help to integrate DTI findings (especially axial and radial changes) and volumetric data on the clinical scenario.
