# Correction: Different Patterns of White Matter Degeneration Using Multiple Diffusion Indices and Volumetric Data in Mild Cognitive Impairment and Alzheimer Patients

**DOI:** 10.1371/annotation/df743c15-c50e-4d00-a24d-510e15f9a73b

**Published:** 2013-06-13

**Authors:** Gilberto Sousa Alves, Laurence O’Dwyer, Alina Jurcoane, Viola Oertel-Knöchel, Christian Knöchel, David Prvulovic, Felipe Sudo, Carlos Eduardo Alves, Letice Valente, Denise Moreira, Fabian Fuβer, Tarik Karakaya, Johannes Pantel, Eliasz Engelhardt, Jerson Laks

Author Fabian Fuβer's name is misspelled. The correct spelling is: Fabian Fußer

There was an error in the list of author affiliations. The full, correct list of authors and affiliations is as follows:

Gilberto Sousa Alves^1,5*^, Laurence O'Dwyer^5^, Alina Jurcoane^6^, Viola Oertel-Knöchel^5^, Christian Knöchel^5^, David Prvulovic^5^, Felipe Sudo^1^, Carlos Eduardo Alves^1^, Letice Valente^1^, Denise Moreira^3,4^, Fabian Fußer^5^, Tarik Karakaya^5^, Johannes Pantel^7^, Eliasz Engelhardt^1,2^, Jerson Laks^1^

**1** Alzheimer's Disease Center - Institute of Psychiatry, Universidade Federal do Rio de Janeiro, Rio de Janeiro, Brazil

**2** Cognitive and Behavior Neurology Unit - Institute of Neurology, Universidade Federal do Rio de Janeiro, Rio de Janeiro, Brazil

**3** Radiology Service - Instituto de Neurologia Deolindo Couto, Universidade Federal do Rio de Janeiro, Rio de Janeiro, Brazil

**4** Procardíaco Hospital, Rio de Janeiro, Brazil

**5** Department of Psychiatry, Psychosomatic Medicine and Psychotherapy, Goethe-University, Frankfurt am Main, Germany

**6** Institute for Neuroradiology, Goethe-University, Frankfurt am Main, Germany

**7** Institute of General Practice, Geriatric Medicine, Goethe-University, Frankfurt am Main, Germany

*E-mail: gsalves123@hotmail.com

